# Supplementary material for: Content-rich biological network constructed by mining PubMed abstracts
Source: BMC Bioinformatics. 2004 Oct 8;5:147. doi: 10.1186/1471-2105-5-147 (PMC528731; doi:10.1186/1471-2105-5-147)
Supplement: Additional File 2 — The original results of the above study (non-essential files are deleted to keep the file size under the limit set by BMC bioinformatics). [file 1471-2105-5-147-S2.bz2 › chilibotAdditionalFile2/dip40/702ID10809785E18035/html/MALG_MALK.html]

 


 **MALG** and **MALK** 
  
Found 36 abstracts in PubMed, retrieved 36.  
 

 What does Google say? 
 PDF only 
| .edu only 

---

**Interactive relationship** (e.g. stimulation, inhibition, etc)

**Neutral relationship**- **MalK**  bound specifically to MalF and  **MalG**  and reconstituted a functional complex.  Ref: 9791180 Mol Microbiol, 1998
- We focused on interactions between the nucleotide binding protein,  **MalK** , and the transmembrane proteins, MalF and  **MalG** .  Ref: 9791180 Mol Microbiol, 1998
- Chemical cross linking detected specific interactions between MalF and  **MalG**  and between MalF and  **MalK** .  Ref: 2026607 J Biol Chem, 1991
- In addition, our results show that they play no role in the interactions with the other proteins of the maltose transport MalF,  **MalG**  or  **MalK**  or chemotaxis Tar systems.  Ref: 2196376 J Mol Biol, 1990
- 1998 Nature London 39 03 707 and are interpreted in favor of a role of the signature sequence in activating the hydrolyzing activity of  **MalK**  upon substrate initiated conformational changes in MalF  **MalG** .  Ref: 10561582 Eur J Biochem, 1999
- We used this approach and limited proteolysis with trypsin to show that binding and hydrolysis of ATP, inducing conformational changes in  **MalK** , modulate its interaction with MalF and  **MalG** .  Ref: 9791180 Mol Microbiol, 1998
- This assay relies on the fact that, when a bifunctional  **MalK**  LacZ hybrid protein is associated with the MalF and  **MalG**  subunits, it is membrane bound.  Ref: 8226895 J Biol Chem, 1993
- To this end, single cysteine residues were introduced into each subunit at several positions and the respective malF and  **malG**  alleles were individually co expressed with each of the  **malK**  alleles.  Ref: 10809785 J Biol Chem, 2000
- The  **MalG**  protein, together with MalF and  **MalK**  proteins, forms a multimeric complex in the membrane consisting of two  **MalK**  subunits for each MalF and  **MalG**  subunit.  Ref: 8437519 Mol Microbiol, 1993
- We have used LacK as a tool to identify sites of interaction of  **MalK**  with the membrane integral components MalF and  **MalG** .  Ref: 8951813 Mol Microbiol, 1996
- This alteration in location suggests that the  **malK**  protein is associated with the inner surface of the cytoplasmic membrane via an interaction with the  **malG**  protein.  Ref: 6778869 J Biol Chem, 1981
- MalF and  **MalG**  are integral transmembrane proteins, while  **MalK**  is associated with the inner aspect of the cytoplasmic membrane via an interaction with  **MalG** .  Ref: 3050132 J Mol Biol, 1988
- The maltose transport complex of Escherichia coli, a member of the ATP binding cassette ABC superfamily, is made up of two nucleotide binding subunits,  **MalK**  2, which hydrolyze ATP with positive cooperativity, and two transmembrane subunits, MalF and  **MalG** .  Ref: 12093921 Proc Natl Acad Sci U S A, 2002
- These results indicate that a putative helical domain in  **MalK** , encompassing residues 89 140, is crucial for a functional, high affinity interaction with MalF and  **MalG** .  Ref: 8951813 Mol Microbiol, 1996
- The results suggest that residues Ala 85, Lys 106, Val 114, and Val 117 in the helical segment of  **MalK** , to different extents, participate in constitution of asymmetric interaction sites with the EAA loops of MalF and  **MalG** .  Ref: 10809785 J Biol Chem, 2000

**Non-interactive relationship** (e.g. studied together, co-existance, homology, etc.)

- In our studies of the oligomerization of this complex, we have shown that the ATP binding component,  **MalK** , forms a dimer in the absence of MalF and  **MalG** .  Ref: 10037713 J Biol Chem, 1999
- The MTC is made up of two integral membrane proteins, MalF and  **MalG** , and a peripheral membrane protein,  **MalK** .  Ref: 10037713 J Biol Chem, 1999
- This uncoupled activity was not due to dissociation of the  **MalK**  ATPase subunit from the integral membrane protein MalF and  **MalG**  subunits.  Ref: 10648525 J Bacteriol, 2000
- First, we optimized the yield in purified complex protein by taking advantage of a newly constructed expression plasmid that carries the  **malK** , malF and  **malG**  genes in tandem orientation.  Ref: 12225853 Biochim Biophys Acta, 2002
- The three membrane associated components of the transport system, the  **MalK** , MalF, and  **MalG**  proteins, have been solubilized from the membrane and maltose transport activity has been reconstituted in proteoliposome vesicles Davidson, A.  Ref: 2026607 J Biol Chem, 1991
- Photolabeling of  **MalK**  required the presence of MalF but not  **MalG** .  Ref: 8226895 J Biol Chem, 1993
- The maltose transport system of E. coli is composed of a periplasmic maltose binding protein MBP, the presumed transmembrane channel made up of MalF and  **MalG**  proteins, and two copies of the ATPase subunit,  **MalK** .  Ref: 8206159 FEBS Lett, 1994
- Analysis of flanking database sequences from M. tuberculosis and M. leprae revealed the presence of malF,  **malG**  and  **malK**  like genes.  Ref: 10817516 Microbios, 2000
- The products of the malF,  **malG** , and  **malK**  genes form a membrane associated complex that catalyzes the hydrolysis of ATP to provide energy for the transport event.  Ref: 1549599 Proc Natl Acad Sci U S A, 1992
- Prior to measuring nucleotide binding, we examined membrane preparations for the presence of each subunit from strains that contained all possible permutations of the three structural genes, malF,  **malG** , and  **malK** .  Ref: 8226895 J Biol Chem, 1993
- LamB, a periplasmic maltose binding protein MalE, MBP and three inner membrane proteins, MalF,  **MalG**  and  **MalK** .  Ref: 3050132 J Mol Biol, 1988
- To study the mechanism of dominance we devised a means of measuring the ability of different wild type and mutant  **MalK**  proteins to interact with the MalF and  **MalG**  subunits.  Ref: 8226895 J Biol Chem, 1993
- ATPase activity was not due to dissociation of the  **MalK**  subunit from the integral membrane proteins MalF and  **MalG**  but could not be further stimulated by trehalose maltose binding protein TMBP, be it the native protein as isolated from T. litoralis or the soluble engineered protein.  Ref: 11453995 Eur J Biochem, 2001
- Cyclodextrin transport is mediated by the products of the cymE, cymF, cymG, cymD, and cymA genes, which are functionally homologous to the malE, malF,  **malG** ,  **malK** , and lamB gene products of Escherichia coli.  Ref: 9573146 J Bacteriol, 1998
- In all experiments, the MalF,  **MalG** , and  **MalK**  proteins behaved as a multiprotein complex.  Ref: 2026607 J Biol Chem, 1991
- one important function of MBP is to transmit a transmembrane signal, through the membrane spanning MalF and  **MalG**  proteins, to the  **MalK**  protein on the other side of the membrane, so that ATP hydrolysis can occur.  Ref: 1549599 Proc Natl Acad Sci U S A, 1992
- Sequence data and genetic evidence indicate that three of them, MalF,  **MalG**  and  **MalK** , are associated with the inner membrane.  Ref: 2233678 Mol Gen Genet, 1990
- Active transport of maltose in Escherichia coli requires the presence of both maltose binding protein MBP in the periplasm and a complex of MalF,  **MalG** , and  **MalK**  proteins FGK2 located in the cytoplasmic membrane.  Ref: 1406246 Mol Microbiol, 1992
- We report the characterization of TrmB, a protein of 3 00 apparent molecular weight, that is involved in the maltose specific regulation of a gene cluster in Thermococcus litoralis, malE malF  **malG**  orf trmB  **malK** , encoding a binding protein dependent ABC transporter for trehalose and maltose.  Ref: 12426307 J Biol Chem, 2003
- The proteins of this system are LamB in the outer membrane, maltose binding protein MBP in the periplasm, and the proteins of the inner membrane complex MalFGK2, composed of one MalF, one  **MalG** , and two  **MalK**  subunits.  Ref: 9401026 J Bacteriol, 1997
- We incubated purified  **MalK**  with inverted membrane vesicles containing MalF and  **MalG** .  Ref: 9791180 Mol Microbiol, 1998
- The maltose transporter contains two transmembrane subunits, MalF and  **MalG** , and two copies of a nucleotide hydrolyzing subunit,  **MalK** .  Ref: 11150310 J Biol Chem, 2001
- We observed a 10 fold increase in the level of transport activity in assays with membrane vesicles when the three membrane associated components of the transport system the MalF,  **MalG** , and  **MalK**  proteins were overproduced.  Ref: 2155217 J Biol Chem, 1990
- We have investigated the proximity of residues in a conserved sequence EAA loop of MalF and  **MalG**  to residues in a helical segment of the  **MalK**  subunits by means of site directed chemical cross linking.  Ref: 10809785 J Biol Chem, 2000
- In Escherichia coli, the binding protein dependent transport system for maltose and maltodextrins is composed of five proteins LamB, MalE, MalF,  **MalG**  and  **MalK**  located in the three layers of the bacterial envelope.  Ref: 8437518 Mol Microbiol, 1993
- A putative helical domain in the  **MalK**  subunit of the ATP binding cassette transport system for maltose of Salmonella typhimurium MalFGK2 is crucial for interaction with MalF and  **MalG** .  Ref: 8951813 Mol Microbiol, 1996
- Each complex contains two  **MalK** , one MalF, and one  **MalG**  proteins, providing two putative sites for ATP hydrolysis.  Ref: 2026607 J Biol Chem, 1991
- Maltose negative mutants fall into two categories with respect to the cellular localization of the  **MalK**  ATPase in the first,  **MalK**  is membrane bound, as in wild type strains, while in the second, it is cytosolic, as in strains deleted in the malF and  **malG**  genes.  Ref: 9214624 EMBO J, 1997
- The binding protein dependent maltose transport system of enterobacteria, a member of the ATP binding cassette ABC transporter superfamily, is composed of two integral membrane proteins, MalF and  **MalG** , and two copies of an ATPase subunit,  **MalK** , which hydrolyze ATP, thus energizing the translocation process.  Ref: 10561582 Eur J Biochem, 1999
- The complex, containing one copy each of the integral membrane proteins  **MalG**  and MalF and two copies of the peripheral cytoplasmic membrane protein  **MalK** , interacts with the periplasmic maltose binding protein to efficiently translocate maltose and maltodextrins across the bacterial cytoplasmic membrane.  Ref: 9573205 J Bacteriol, 1998
- The formation of a  **MalK**  dimer in the absence of MalF and  **MalG**  may represent an initial step in the assembly pathway of the MTC.  Ref: 10037713 J Biol Chem, 1999
- In mutant strains which lack another component of the transport system, the  **malG**  protein, the  **malK**  protein is located in the cytoplasm.  Ref: 6778869 J Biol Chem, 1981
- In addition to the water soluble maltose binding protein, the system comprises three membrane proteins, MalF,  **MalG** , and  **MalK** , which form a heterotetrameric complex FGK2 in the cytoplasmic membrane.  Ref: 8226895 J Biol Chem, 1993
- The binding protein dependent maltose transport system of enterobacteria MalFGK 2, a member of the ATP binding cassette ABC transporter superfamily, is composed of two integral membrane proteins, MalF and  **MalG** , and of two copies of an ATPase subunit,  **MalK** , which hydrolyze ATP, thus energizing the translocation process.  Ref: 10809785 J Biol Chem, 2000
